# Supplementary material for: Longitudinal Trajectory Modeling to Assess Adherence to Sacubitril/Valsartan among Patients with Heart Failure
Source: Pharmaceutics. 2023 Nov 1;15(11):2568. doi: 10.3390/pharmaceutics15112568 (PMC10674925; doi:10.3390/pharmaceutics15112568)
Supplement: Supplementary file 1 [file pharmaceutics-15-02568-s001.zip › pharmaceutics-2651552-supplementary.pdf]

pharmaceutics

Supplementary Material

Figure S1. Study flow chart

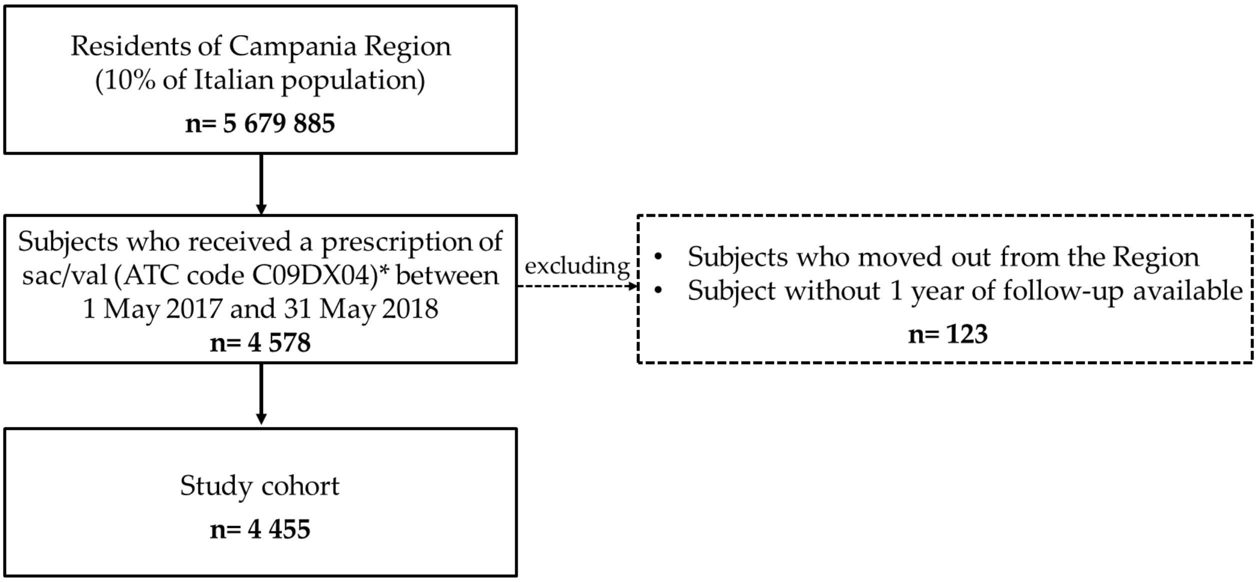

*Notes: Sac/val received approval for the treatment of chronic heart failure (HF) with reduced ejection fraction (HFrEF), specifically in cases where the left ventricular ejection fraction (LVEF) is ⩽40%. Therefore, the prescription of sac/val inherently implies the presence of HF. Furthermore, sac/val was introduced to the Italian market in May 2017. Given that our recruitment period commenced on January 1, 2017, all subjects within our study cohort initiated sac/val treatment, rendering the application of a wash-out period unnecessary for these reasons.

Table S1. Baseline patient's characteristics stratified by Sac/Val index dosage (N = 4 455)

**Characteristics**	**Low-dosage (24mg/26mg)**	**Medium-dosage (49mg/51mg)**	**High-dosage (97mg/103mg)**
**Total ° , N (%)**	**2 941 (66)**	**1 306 (29.3)**	**208 (4.7)**
**Gender , N (%)**			
Female	876 (29.8)	407 (31.2)	50 (24)
Male	2 057 (69.9)	897 (68.7)	156 (75)
**Age, Mean (SD)**	**69.5±11.9**	**68.8±12.0**	**65.6±12.5**
**Age groups, N (%)**			
0-25 y	10 (0.3)	6 (0.5)	1 (0.5)
26-50 y	192 (6.5)	87 (6.7)	26 (12.5)
51-75 y	1 733 (58.9)	788 (60.3)	131 (63)
over 76 y	998 (33.9)	423 (32.4)	48 (23.1)
**Polypharmacy,* N (%)**			
No polypharmacy (1-4 drugs)	895 (30.4)	399 (30.6)	69 (33.2)
Polypharmacy (5-9 drugs)	943 (32.1)	404 (30.9)	63 (30.3)
Excessive polypharmacy (≥10 drugs)	1 081 (36.8)	498 (38.1)	75 (36.1)
**ACCI score, Mean (SD)**	**8.1 (6.5)**	**7.7 (5.8)**	**6.9 (4.8)**
**Hospital admission,* Mean (SD)**	**1.7 (1.1)**	**1.7 (1.2)**	**1.6 (1.0)**
Had ≥1 hospitalization for HF, N (%)	1 061 (36.1)	364 (27.9)	64 (30.8)
Had ≥2 hospitalization for other causes, N (%)	587 (20)	252 (19.3)	36 (17.3)
**Medication HF-related,* N (%)**			
Beta blocking agents	2 603 (88.5)	1 125 (86.1)	177 (85.1)
Antithrombotic agents	2 479 (84.3)	1 085 (83.1)	169 (81.3)
Agents acting on the renin-angiotensin system	2 338 (79.5)	1 111 (85.1)	176 (84.6)
Diuretics	2 381 (81)	1 005 (77)	145 (69.7)
**Other medications,* N (%)**			
Lipid modifying agents	1 964 (66.8)	915 (70.1)	139 (66.8)
Cardiac therapy	1 560 (53)	706 (54.1)	98 (47.1)
Drugs for obstructive airway diseases	1 275 (43.4)	538 (41.2)	79 (38)
Drugs used in diabetes	1 023 (34.8)	490 (37.5)	70 (33.7)
**Cardiovascular comorbidities,* N (%)**			
Cardiomyopathy	293 (10)	107 (8.2)	22 (10.6)
CCS	199 (6.8)	118 (9)	22 (10.6)
Hypertension	148 (5)	53 (4.1)	20 (9.6)
CCS with STEMI	129 (4.4)	46 (3.5)	4 (1.9)
CCS with NSTEMI	109 (3.7)	50 (3.8)	5 (2.4)
Atrial fibrillation	107 (3.6)	38 (2.9)	4 (1.9)
**Other comorbidities,* N (%)**			
Diabetes	349 (11.9)	141 (10.8)	16 (7.7)
Chronic kidney disease	143 (4.9)	56 (4.3)	7 (3.4)
Respiratory failure	97 (3.3)	31 (2.4)	4 (1.9)

° Percentage calculated on the total of subjects analyzed * Conditions occurring one year prior to the index date of initiation of Sac/Val therapy
Notes: Low-dose: sacubitril 24mg / valsartan 26mg; Medium-dose: sacubitril 49mg / valsartan 51mg; High-dose: sacubitril 97mg / valsartan 103mg
Abbreviations: CCS, Chronic coronary syndrome; ICH, Ischemia; HF, Heart failure; IQR, Interquartile range; NSTEMI, non-ST elevation myocardial infarction; SD, Standard deviation; STEMI, ST elevation myocardial infarction.

Figure S2. Calinski & Harabasz (CH) sorted criterion chosen for the selection of the four adherence groups

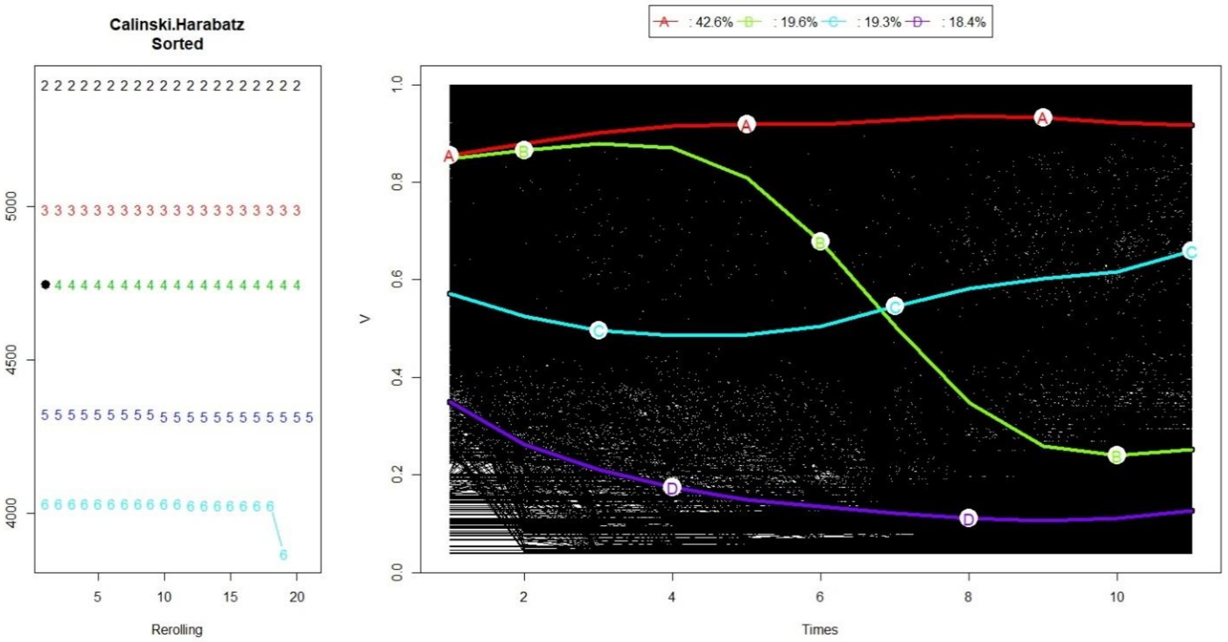

pharmaceutics MDPI

Table S2. Logistic regression models of predictors of adherence trajectories

Logistic regression model	Group A High Adherence		Group B Partial drop-off		Group C Moderate adherence		Group D Low adherence	
	Adjusted OR (95% CI)	*p-value*	Adjusted OR (95% CI)	*p-value*	Adjusted OR (95% CI)	*p-value*	Adjusted OR (95% CI)	*p-value*
**Gender**								
Female (vs male)	1.064 (1.053-1.074)	*<0.001*	0.913 (0.902-0.925)	*<0.001*	1.239 (1.222-1.255)	*<0.001*	0.815 (0.805-0.825)	*<0.001*
**Polypharmacy**								
Polypharmacy (vs no polypharmacy)	1.052 (1.039-1.066)	*<0.001*	1.194 (1.175-1.214)	*<0.001*	0.943 (0.928-0.959)	*<0.001*	0.827 (0.813-0.84)	*<0.001*
Excessive polypharmacy (vs no polypharmacy)	1.041 (1.029-1.054)	*<0.001*	1.184 (1.166-1.202)	*<0.001*	0.913 (0.899-0.926)	*<0.001*	0.877 (0.864-0.89)	*<0.001*
**Hospital admission**								
Had ≥1 hospitalization for HF	0.981 (0.971-0.99)	*<0.001*	0.9 (0.888-0.911)	*<0.001*	0.98 (0.968-0.993)	*0.002*	1.165 (1.151-1.179)	*<0.001*
Had ≥2 hospitalization for other causes	0.781 (0.771-0.791)	*<0.001*	1.124 (1.107-1.141)	*<0.001*	0.851 (0.838-0.865)	*<0.001*	1.481 (1.459-1.503)	*<0.001*
**Medications HF-related**								
Beta blocking agents	1.003 (0.988-1.019)	*0.675*	1.021 (1.002-1.041)	*0.035*	0.988 (0.969-1.008)	*0.231*	0.986 (0.967-1.005)	*0.142*
Antithrombotic agents	0.995 (0.979-1.012)	*0.577*	1.009 (0.988-1.031)	*0.403*	1.013 (0.992-1.035)	*0.226*	0.986 (0.965-1.007)	*0.182*
Agents acting on the renin-angiotensin system	1.016 (0.999-1.032)	*0.060*	1.009 (0.989-1.03)	*0.390*	1.001 (0.981-1.021)	*0.937*	0.967 (0.947-0.986)	*0.001*
Diuretics	1.041 (1.024-1.058)	*<0.001*	0.991 (0.971-1.012)	*0.400*	0.967 (0.947-0.987)	*0.002*	0.979 (0.959-0.999)	*0.036*
**Other medications**								
Lipid modifying agents	0.995 (0.977-1.013)	*0.565*	0.995 (0.973-1.017)	*0.629*	0.991 (0.969-1.014)	*0.452*	1.022 (1-1.044)	*0.054*
Cardiac therapy	0.969 (0.949-0.989)	*0.003*	0.971 (0.946-0.997)	*0.032*	1.146 (1.117-1.176)	*<0.001*	0.939 (0.915-0.965)	*<0.001*
Drugs for obstructive airway diseases	0.945 (0.916-0.975)	*<0.001*	1.212 (1.168-1.257)	*<0.001*	0.922 (0.886-0.959)	*<0.001*	0.966 (0.93-1.004)	*0.080*
Drugs used in diabetes	1.085 (1.062-1.109)	*<0.001*	0.937 (0.911-0.964)	*<0.001*	1.002 (0.974-1.03)	*0.907*	0.933 (0.908-0.96)	*<0.001*
**Cardiovascular comorbidities**								
Cardiomyopathy	1.054 (1.04-1.068)	*<0.001*	1.018 (1.001-1.036)	*0.042*	0.912 (0.896-0.928)	*<0.001*	0.989 (0.972-1.006)	*0.190*
CCS	0.469 (0.434-0.507)	*<0.001*	0.962 (0.882-1.05)	*0.388*	2.514 (2.341-2.701)	*<0.001*	0.983 (0.903-1.071)	*0.702*
Hypertension	0.787 (0.765-0.81)	*<0.001*	1.34 (1.297-1.384)	*<0.001*	1.036 (1-1.073)	*0.047*	1.01 (0.976-1.045)	*0.566*
CCS with STEMI	0.893 (0.867-0.919)	*<0.001*	1.11 (1.072-1.149)	*<0.001*	0.89 (0.857-0.924)	*<0.001*	1.18 (1.141-1.221)	*<0.001*
Atrial fibrillation	1.109 (1.093-1.126)	*<0.001*	1.012 (0.993-1.03)	*0.220*	0.99 (0.972-1.009)	*0.302*	0.849 (0.833-0.865)	*<0.001*
CCS with NSTEMI	1.057 (1.026-1.09)	*<0.001*	1.529 (1.478-1.582)	*<0.001*	0.904 (0.869-0.94)	*<0.001*	0.589 (0.564-0.616)	*<0.001*
**Other comorbidities**								
Diabetes	0.98 (0.963-0.996)	*0.017*	0.853 (0.835-0.872)	*<0.001*	1.212 (1.187-1.236)	*<0.001*	0.992 (0.972-1.013)	*0.448*
Chronic kidney disease	0.802 (0.782-0.823)	*<0.001*	0.867 (0.839-0.895)	*<0.001*	1.595 (1.55-1.641)	*<0.001*	0.965 (0.936-0.995)	*0.023*
Respiratory failure	0.989 (0.968-1.011)	*0.311*	0.966 (0.94-0.993)	*0.014*	0.97 (0.944-0.998)	*0.034*	1.079 (1.051-1.107)	*<0.001*
